# Assessing Conformance with Benford’s Law: Goodness-Of-Fit Tests and Simultaneous Confidence Intervals

**DOI:** 10.1371/journal.pone.0151235

**Published:** 2016-03-28

**Authors:** M. Lesperance, W. J. Reed, M. A. Stephens, C. Tsao, B. Wilton

**Affiliations:** 1 Department of Mathematics and Statistics, University of Victoria, Victoria, Canada; 2 Simon Fraser University, Burnaby, Canada; 3 Camosun College, Victoria, Canada; Ohio State University College of Medicine, UNITED STATES

## Abstract

Benford’s Law is a probability distribution for the first significant digits of numbers, for example, the first significant digits of the numbers 871 and 0.22 are 8 and 2 respectively. The law is particularly remarkable because many types of data are considered to be consistent with Benford’s Law and scientists and investigators have applied it in diverse areas, for example, diagnostic tests for mathematical models in Biology, Genomics, Neuroscience, image analysis and fraud detection. In this article we present and compare statistically sound methods for assessing conformance of data with Benford’s Law, including discrete versions of Cramér-von Mises (CvM) statistical tests and simultaneous confidence intervals. We demonstrate that the common use of many binomial confidence intervals leads to rejection of Benford too often for truly Benford data. Based on our investigation, we recommend that the CvM statistic $U_{d}^{2}$, Pearson’s chi-square statistic and 100(1 − *α*)% Goodman’s simultaneous confidence intervals be computed when assessing conformance with Benford’s Law. Visual inspection of the data with simultaneous confidence intervals is useful for understanding departures from Benford and the influence of sample size.

## Introduction

Benford’s Law is a probability distribution for the first significant digit (FSD) of numbers, for example, the FSD of the numbers 871 and 0.0561 are 8 and 5 respectively. The law is based on the empirical observation that for many sets of numerical data the FSD is not uniformly distributed, as might naively be expected, but rather follows a logarithmic distribution, that is, for first digit *D*_1_,

$$\begin{array}{c} \text{Pr}\left(D_{1}=d\right)=\log_{10}\left[1+1/d\right],\;\text{for}\;d=1,2,\cdots ,9. \end{array}$$ (1)

For example, the probability that the first digit is 3 is log_10_[1 + 1/3] ≈ 0.1249. The law is remarkable because many types of data are considered to be consistent with Benford’s Law. The Benford Online Bibliography [1] is a large database of papers, books, websites, etc. which apply Benford’s Law in diverse areas, from diagnostic tests for mathematical models in Biology, Genomics, Neuroscience, to image analysis and fraud detection by the U.S. Internal Revenue Service, and two recent books [2, 3] also bear testimony to the popularity of the law in many fields.

To demonstrate conformance with Benford’s Law, many authors use simple statistical methodology: visual plots, Pearson’s chi-square test and individual confidence intervals for digit probabilities based on the binomial distribution. These methods may be inefficient, inaccurate, or lacking in power to detect reasonable departures from (alternatives to) Benford’s Law. In particular, methods based on individual confidence intervals do not take into consideration the phenomenon of multiple comparisons. For example, the **joint** confidence level for nine binomial 100(1 − *α*)% confidence intervals computed using the observed proportions of leading digits 1 through 9 in a sample of numbers may be very different from 100(1 − *α*)%, the analyst’s intended confidence level, and the problem is magnified if the first two or more digits are considered.

Often data sets are large, and Miller’s (Chapter 1, 2015) [4] remark concerning conformance with Benford’s Law, “It is a non-trivial task to find good statistical tests for large data sets”, is pertinent. In this article we present and compare statistically sound methods for assessing conformance of data to Benford’s Law for medium to large data sets. We investigate the likelihood ratio test for the most general alternative, three tests based on Cramér-von Mises statistics for discrete distributions, Pearson’s chi-square statistic and simultaneous confidence interval procedures for assessing compliance with the set of Benford probabilities.

Because Benford’s Law is of wide application and general interest, we first present a brief description of the law. This is followed by sections on the goodness-of-fit tests and simultaneous confidence intervals for multinomial probabilities. Comparisons of the power of the procedures to detect various plausible alternatives are provided as well as examples from Genomics and Finance. The final section concludes with a discussion of the results. An R [5] package for these methods is freely available.

## Benford’s Law

Benford’s Law is based on the empirical observation that for many sets of numerical data, the first significant (or leading) digits follow a logarithmic distribution. For the first *m* digits, *D*_1_,*D*_2_, …,*D*_*m*_,

$$\begin{array}{c} \text{Pr}\left(D_{1}=d_{1},D_{2}=d_{2},\cdots ,D_{m}=d_{k}\right)=\log_{10}\left[1+{\left(\sum_{j=1}^{m}d_{j}\times 10^{m-j}\right)}^{-1}\right], \end{array}$$ (2)

for *d*_1_ = 1, 2, …, 9 and *d*_2_, …, *d*_*m*_ = 0, 1, …, 9, so that, for example, the probability that the first two digits are 30 is log_10_[1 + (30)^−1^] ≈ 0.01424 and the probability that the first three digits are 305 is log_10_[1 + (305)^−1^] ≈ 0.00142. This closely agrees with empirical distributions of first digits in much tabular data: for example, [6] considered areas of rivers, American League baseball statistics, atomic weights of elements and numbers appearing in *Reader’s Digest* articles.

There have been many attempts to explain Benford’s Law—see [2, 3, 7–9] for reviews of these. One of the most convincing explanations is that put forward by Hill [8], who demonstrated that if numbers are generated by first selecting probability distributions at random and then choosing and combining random samples from said distributions, the distribution of FSDs will converge to Benford’s Law provided that the sampling is unbiased with regard to scale or base [2]. Thus, even if tabular data come from many sources, one might expect the empirical first digit frequencies to closely follow Benford’s Law. Other explanations are provided in the books [2, 3] and include: spread, geometric, scale-invariance and Central Limit Theorem explanations.

Not all datasets conform to Benford’s Law. For example, it does not hold for tables of (uniformly distributed) random numbers, nor for numbers in telephone directories, nor for dates (*mm/dd/yy* or *dd/mm/yy*). Rodriguez (2004) [10] demonstrates that Benford’s Law is inadequate when data are drawn from commonly used distributions, including the standard normal, Cauchy and exponential distributions. He does show, however, that the Lognormal distribution yields FSD probabilities arbitrarily close to Benford as the log-scale variance increases.

## Likelihood ratio and Pearson’s chi-square tests for Benford’s Law

Likelihood ratio tests are generally powerful tests [11] and are often the tests of choice of statisticians. Given the FSDs of a set of *n* entries in a set of data, we test whether they are compatible with Benford’s Law Eq (1). That is, we test the null hypothesis for the first digit probabilities, *p*_*i*_ ≡ *Pr*(*D*_1_ = *i*),

$$\begin{array}{c} H_{0}:p_{i}=\log_{10}\left(1+1/i\right),\;\;\;\;\;\text{for}\;i=1,2,\cdots 9 \end{array}$$

against the broadest alternative hypothesis,

$$\begin{array}{c} H_{1}:p_{1}\geq 0,\cdots ,p_{9}\geq 0;\;\;\;\sum_{i=1}^{9}p_{i}=1. \end{array}$$

With first digit frequencies, *f*_*i*_, and observed proportions, $\hat{p_{i}}=f_{i}/n$, *i* = 1, 2…, 9, the likelihood ratio (LR) statistic Λ for testing $H_{0}$ *vs.* $H_{1}$ is given by

$$\begin{array}{c} -2\ln\Lambda =2\sum_{i=1}^{9}n\hat{p_{i}}\left\{\ln\;\frac{\hat{p_{i}}}{p_{i}}\right\}, \end{array}$$

which asymptotically follows a $\chi_{\left(8\right)}^{2}$ distribution, where ln is natural log. The LR test is asymptotically equivalent to Pearson’s chi-square statistic,

$$\begin{array}{c} X^{2}=\sum_{i=1}^{9}\frac{{\left(f_{i}-np_{i}\right)}^{2}}{np_{i}}=n\sum_{i=1}^{9}\frac{{\left({\hat{p}}_{i}-p_{i}\right)}^{2}}{p_{i}}. \end{array}$$ (3)

## Tests based on Cramér-von Mises statistics

In this section we consider omnibus goodness-of-fit tests based on the Cramér-von Mises type (CvM) statistics for discrete distributions [12, 13]. Specifically we consider statistics $W_{d}^{2}$, $U_{d}^{2}$ and $A_{d}^{2}$ which are analogues of, respectively, the Cramér-von Mises, Watson and Anderson-Darling statistics, widely used for testing goodness of fit for continuous distributions. These discrete CvM statistics have been shown to have greater power than Pearson’s chi-square statistic when testing for the grouped exponential distribution and the Poisson distribution [14–16].

As above, we test Benford’s Law against the most general alternative hypothesis, $H_{1}$. Let $S_{i}=\sum_{j=1}^{i}{\hat{p}}_{j}$ and $T_{i}=\sum_{j=1}^{i}p_{j}$ denote the cumulative observed and expected proportions, and *Z*_*i*_ = *S*_*i*_ − *T*_*i*_. Note that *Z*_*i*_ is the difference between the empirical and null cumulative distribution functions on which the CvM statistics are based. Define weights *t*_*i*_ = (*p*_*i*_ + *p*_*i* + 1_)/2 for *i* = 1, …, 8 and *t*_9_ = (*p*_9_ + *p*_1_)/2 and define the weighted mean of the deviations *Z*_*i*_ as $\bar{Z}=\sum_{i=1}^{9}t_{i}Z_{i}$. The CvM statistics are defined as follows [13]

$$\begin{array}{cc} W_{d}^{2} & =n\sum_{i=1}^{9}Z_{i}^{2}t_{i};\\ U_{d}^{2} & =n\sum_{i=1}^{9}{\left(Z_{i}-\bar{Z}\right)}^{2}t_{i};\\ A_{d}^{2} & =n\sum_{i=1}^{9}Z_{i}^{2}t_{i}/\left\{T_{i}\left(1-T_{i}\right)\right\}. \end{array}$$

Note that since *Z*_9_ = 0 the last term in $W_{d}^{2}$ is zero. The last term in $A_{d}^{2}$ is of the form 0/0, and is set equal to zero.

The CvM type statistics defined here take into account the order of the cells (or, digits here) in contrast to Pearson’s statistic, *X*^2^, which does not. However, if the order of the cells is completely reversed, the values of the statistics are unaltered. Further, the statistic $U_{d}^{2}$ is invariant to the choice of the origin for the hypothesized discrete distribution [13].

Under the null hypothesis, the asymptotic distribution of the CvM statistics is a linear combination of independent $\chi_{\left(1\right)}^{2}$ random variables. Asymptotic percentage points (or critical values) for the CvM statistics under the null are in Table 1 and R code for computing p-values for these statistics is available. Upper-tail probabilities for the asymptotic distribution can be obtained using a numerical method due to Imhof [17, 18] or more crudely using a chi-square approximation. Imhof’s method requires numerical integration in one dimension of a closed form expression, whereas the chi-square approximation is faster to compute since it only requires the first three cumulants of the statistic in question.

**Table 1:** Asymptotic percentage points for Cramer-von Mises statistics.

	*α*					
	0.500	0.250	0.100	0.050	0.025	0.010
$W_{d}^{2}$	0.110	0.206	0.351	0.471	0.597	0.768
$U_{d}^{2}$	0.066	0.108	0.163	0.205	0.247	0.304
$A_{d}^{2}$	0.596	1.060	1.743	2.304	2.890	3.688
Pearson’s *X*^2^	7.344	10.219	13.362	15.507	17.535	20.090

Asymptotic percentage points for Cramer-von Mises statistics are given for testing the null hypothesis of Benford for various values of *α*.

## Simultaneous confidence intervals for multinomial probabilities

Confidence intervals provide more information about departures from Benford’s Law than do p-values for goodness-of-fit. Ideally, we wish to compute a 100(1 − *α*)% set of confidence intervals, with overall confidence level 100(1 − *α*)%, for the nine, or more generally, *k*, digit probabilities using the observed digit frequencies *f*_1_, *f*_2_, …, *f*_*k*_. If all of the *k* confidence intervals cover all of the Benford probabilities, then the data are deemed to be consistent with Benford’s Law at the 100(1 − *α*)% level. If they do not, we can easily determine for which digits departures occur and investigate further. The widths of the confidence intervals also clearly indicate the amount of information in the data which is related to the sample size, *n*. The larger *n*, the narrower the confidence intervals and indeed, extremely narrow confidence intervals that do not all cover all of the Benford probabilities may not be considered as practically significant departures from Benford’s Law.

One approach that is commonly used to generate confidence intervals for multinomial probabilities is to compute, for each cell/digit in turn, a 100(1 − *α*)% (approximate) binomial confidence interval for that digit frequency versus all of the others, i.e. $\hat{p_{i}}\;\bar{+}\;z_{\alpha /2}\sqrt{\frac{\hat{p_{i}}\left(1-\hat{p_{i}}\right)}{n}}$. This procedure uses many (*k* here) single 100(1 − *α*)% confidence intervals and is problematic since the probability that all of these confidence intervals **simultaneously** contain the population proportions is not (1 − *α*), and it can be as small as (1 − *kα*) by the Bonferroni inequality. To remedy this, we use *simultaneous* 100(1 − *α*)% confidence intervals constructed so that the probability that every one of the intervals will contain the corresponding population proportion is (approximately) (1 − *α*).

Several simultaneous confidence intervals for multinomial proportions have been proposed in the literature. We consider six techniques, ordered by date of publication, and present their formulae and some background below. Let **f** = (*f*_1_, …, *f*_*k*_)^*T*^ be the vector of observed cell frequencies, $\chi_{\nu ,\alpha }^{2}$ be the upper *α*th quantile of the chi-square distribution with *ν* degrees of freedom and *z*_*α*_ be the upper *α*th quantile of the standard normal distribution. R code for computing the following simultaneous confidence intervals is available.

1. Quesenberry and Hurst [**Ques**] [19]: The Ques simultaneous confidence intervals are constructed so that the probability that all of them cover the corresponding Benford’s probabilities is at least (1 − *α*), i.e. they are conservative. The theory for the construction is based on the asymptotic *χ*^2^ distribution of Pearson’s chi-square statistic Eq (3) and are recommended when the smallest expected frequency, *np*_*i*_, is at least 5.
  $$\begin{array}{cc} {\;S}_{1}\left(f\right) & =\left\{p\;|\;p_{i}\in \frac{A+2f_{i}\bar{+}{\left\{A\left[A+4f_{i}\left(n-f_{i}\right)/n\right]\right\}}^{1/2}}{2(n+A)}\text{,}\;\right.\\ & i=1,2,\ldots k\} \end{array}$$
  where $A=\chi_{k-1,\alpha }^{2}$.
2. Goodman [**Good**][20]: The Good simultaneous intervals modify the Ques intervals, replacing *A* with *B* to obtain typically shorter, and thus less conservative, intervals.
  $$\begin{array}{cc} S_{2}\left(f\right) & =\left\{p\;|\;p_{i}\in \frac{B+2f_{i}\bar{+}{\left\{B\left[B+4f_{i}\left(n-f_{i}\right)/n\right]\right\}}^{1/2}}{2(n+B)}\text{,}\;\right.\\ & i=1,2,\ldots k\}\;\text{where}\;k\neq 2, \end{array}$$
  and where *B* = $\chi_{1,\alpha /k}^{2}$.
3. Bailey angular transformation [**Bang**] [21]: Bailey modifies the Good simultaneous intervals, incorporating transformations of the observed frequencies which are known to be more nearly normally distributed, for large *n*, than the frequencies themselves. The first modification uses the arcsin-square-root transformation which is a variance stabilizing transformation for binomial data. We do not incorporate corrections for continuity since sample sizes are generally large in Benford’s Law studies.
  $$\begin{array}{cc} S_{3}\left(f\right) & =\left\{p\;|\;\;p_{i}\in {\left\{\sin\left[\sin^{-1}\left(\sqrt{\frac{f_{i}+\frac{3}{8}}{n+\frac{3}{4}}}\right)\bar{+}\sqrt{\frac{B}{4n+2}}\right]\right\}}^{2}\right.\text{,}\;\\ & i=1,2,\ldots m\} \end{array}$$
4. Bailey square root transformation [**Bsqrt**] [21]: Bsqrt simultaneous intervals incorporate a square-root transformation which is a variance stabilizing transformation for Poisson variates.
  $$\begin{array}{cc} S_{4}\left(f\right) & =\left\{p\;|\;p_{i}\in \{{\left.\sqrt{\frac{f_{i}+\frac{3}{8}}{n+\frac{1}{8}}}\bar{+}\sqrt{C\left(C+1-\frac{f_{i}+\frac{3}{8}}{n+\frac{1}{8}}\right)}\right\}}^{2}/{\left(C+1\right)}^{2}\text{,}\;\right.\\ & i=1,2,\ldots m\}, \end{array}$$
  where *C* = *B*/(4*n*).
5. Fitzpatrick and Scott [**Fitz**] [22]: Fitzpatrick and Scott begin with the simple, approximate binomial confidence intervals with $\hat{p_{i}}$ replaced by 1/2 in the standard error, i.e. $\hat{p_{i}}\;\bar{+}\;z_{\alpha /2}\sqrt{\frac{1}{4n}}$. They show that a lower bound for the simultaneous coverage probability of the *k* intervals is (1 − 2*α*) for small *α*. Therefore, their 100(1 − *α*)% intervals take the form:
  $$\begin{array}{c} S_{5}\left(f\right)=\left\{p\;|\;p_{i}\in {\hat{p}}_{i\;}\bar{+}\frac{D}{2\sqrt{n}}\text{,}\;i=1,2,\ldots k\right\}, \end{array}$$
  where *D* = *z*_*α*/4_.
6. Sison and Glaz [**Sison**][23]: The Sison simultaneous confidence intervals are based on a relatively complex approximation for the probabilities that multinomial frequencies lie within given intervals. This procedure does not have a closed form and must be implemented using a computer. Let *V*_*i*_ and *Y*_*i*_, *i* = 1, 2, …, *k*, be independent Poisson random variables with mean *f*_*i*_ and its truncation to [*f*_*i*_ − *τ*, *f*_*i*_ + *τ*], respectively, where *τ* is some constant. Let $f_{1}^{*},f_{2}^{*},$ …, $f_{m}^{*}$ be the cell frequencies in a sample of *n* observations from a multinomial distribution with cell probabilities (*f*_1_/*n*, …, *f*_*m*_/*n*). Define
  $$\begin{array}{c} \mu_{1}=E\left(Y_{i}\right),\sigma_{i}^{2}=V\left(Y_{i}\right),\mu_{\left(r\right)}=E\left[Y_{i}\left(Y_{i}-1\right)\ldots \left(Y_{i}-r+1\right)\right], \end{array}$$
  $$\begin{array}{c} \mu_{r,i}=E{\left(Y_{i}-\mu_{i}\right)}^{r},\gamma_{1}=\frac{\frac{1}{m}\sum_{i=1}^{m}\mu_{3,i}}{\sqrt{m}{\left(\frac{1}{m}\sum_{i=1}^{m}\sigma_{i}^{2}\right)}^{\frac{3}{2}}},\gamma_{2}=\frac{\frac{1}{m}\sum_{i=1}^{m}\mu_{4,i}-3\sigma_{i}^{4}}{\sqrt{m}{\left(\frac{1}{m}\sum_{i=1}^{m}\sigma_{i}^{2}\right)}^{2}}, \end{array}$$
  $$\begin{array}{c} f_{e}\left(x\right)=\left(\frac{1}{\sqrt{2\pi }}e^{\frac{-x^{2}}{2}}\right)1+\frac{\gamma_{1}}{6}\left(x^{3}-3x\right)+\frac{\gamma_{2}}{24}\left(x^{4}-6x^{2}+3\right)+\frac{\gamma_{1}^{2}}{72}\left(x^{6}-15x^{4}+45x^{2}-15\right)\}, \end{array}$$
  $$\begin{array}{c} \nu \left(\tau \right)=\frac{n!}{n^{n}e^{-n}}\left\{\underset{i=1}{\overset{m}{\Pi }}P_{r}\left[n_{i}-\tau \leq V_{i}\leq n_{i}+\tau \right]\right\}f_{e}\left(\frac{n-\sum_{i=1}^{m}\mu_{i}}{\sqrt{\sum_{i=1}^{m}\sigma_{i}^{2}}}\right)\frac{1}{\sqrt{\sum_{i=1}^{m}\sigma_{i}^{2}}}. \end{array}$$
  The Sison and Glaz interval has the following form:
  $$\begin{array}{c} S_{6}\left(f\right)=\left\{p\;|\;\frac{f_{i}}{n}-\frac{\tau }{n}\leq p_{i}\leq \frac{f_{i}}{n}+\frac{\tau +2\gamma }{n}\text{,}\;i=1,2,\ldots m\right\}, \end{array}$$
  where the integer *τ* satisfies the condition *ν*(*τ*) < 1 − *α* < *ν*(*τ* + 1), and *γ* = (1 − *α*) − *ν*(*τ*)/*ν*(*τ* + 1) − *ν*(*τ*).
7. Univariate approximate Binomial confidence intervals.
  $$\begin{array}{c} S_{7}\left(f\right)=\left\{p\;|\;p_{i}\in {\hat{p}}_{i}\;\bar{+}\;G\;\sqrt{\frac{{\hat{p}}_{i}\left(1-{\hat{p}}_{i}\right)}{n}}\text{,}\;i=1,2,\ldots k\right\}, \end{array}$$
  where *G* = *z*_*α*/2_.

## Simulation Study

We investigated the finite sample behaviour of the test statistics and confidence intervals using a simulation study assuming several different alternative distributions. The simulation results, size (proportion of tests rejected when the data are truly Benford) and power (proportion of tests rejected when the data are truly not Benford), are compared.

We considered three sample sizes, *n* = 100, *n* = 1,000 and *n* = 10,000. Ten thousand (N = 10,000) random samples were generated using each of the distributions listed in Table 2, which are alternative distributions that could be reasonably expected to arise in practice. The continuous distributions listed are commonly used and Rodriguez (2004) [10] studies and tabulates the first significant digit probabilities for each of these distributions. The “contaminated” distributions arise from contaminating one digit by *γ*, the amount specified in the table. Each digit is contaminated in turn, increasing that digit’s Benford probability by *γ*, then the remaining digit probabilities are scaled so that all sum to one. This type of distribution was found to arise, in practice, for example when one specific accounting transaction had been processed many times. The *Generalized Benford’s Law* [24] for the first digit, *D*_1_ is,

$$\begin{array}{c} \text{Pr}\left(D_{1}=d\right)=\frac{d^{-\gamma }-{\left(d+1\right)}^{-\gamma }}{1-10^{-\gamma }},\;\;\;\;\;\text{for}\;d=1,2,\cdots 9\text{,}\;\gamma \in ℜ, \end{array}$$ (4)

which was found to approximate the distribution of first digits for southern California earthquake magnitudes. The Uniform/Benford mixture distribution could arise if a proportion, *γ* of data is generated (possibly fabricated) from a first digit uniform distribution while the remainder of the data conforms to Benford.

**Table 2:** Distributions used in the simulation study.

Distribution	Parameter values	Notes
Benford		
Discrete Uniform	*p*_*i*_ = 1/9 for *i* = 1, …9	
Continuous Uniform(*a*, *b*)	(*a*, *b*) ∈ {(0, 10), (0, 43), (0, 76)}	
Normal	(*μ*, *σ* ^2^) ∈ {(0, 1), (13, 400)}	
Exponential	(rate) ∈ {0.2, 1.0}	
Cauchy	(scale) ∈ {.5, 1.0}	
Lognormal	(*μ*log, *σ* ^2^log) ∈ {(0, 1), (2, 1), (2, 9)}	1
Contaminated Benford	*γ* ∈ {.01, .02, .03, .04, .05, .06}	2
Generalized Benford Eq (4)	*γ* ∈ {−1, −.9, …, , .9, 1}	
Uniform/Benford mixture	*γ* ∈ {.1, .2, .3, .4, .5}	3

^1^ (*μ*log, *σ*^2^log) are the mean and variance of the distribution of *X* = ln*Y* where *Y* is Lognormal.

^2^ each *p*_*i*_ in turn is increased by *γ*; the remaining 8 digits are rescaled to sum to one

^3^ *γ* is the proportion Uniform

### Results: Test Statistics

**Fig 1:**
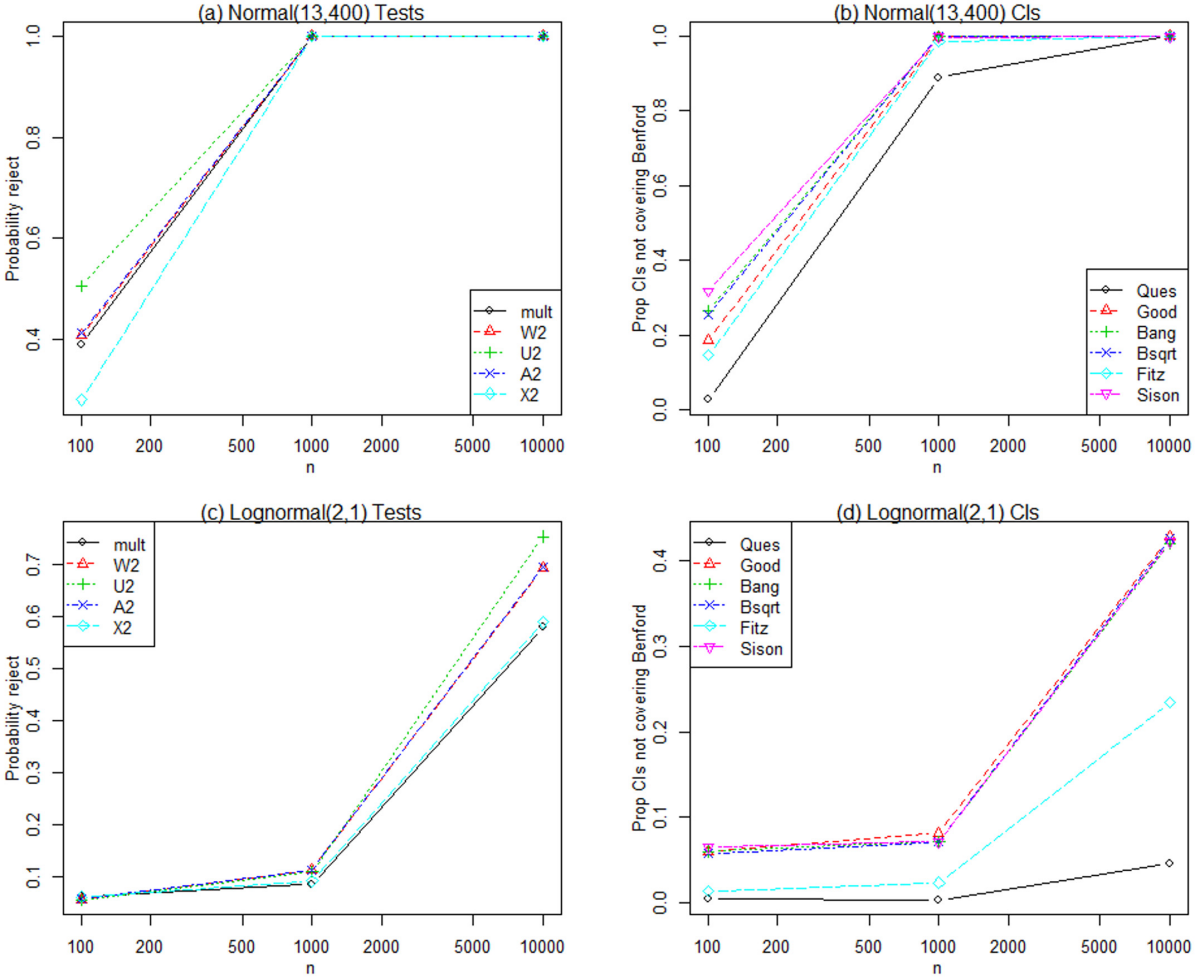
Normal(13,400) and Lognormal(2,1) results. Simulated power for the tests and simultaneous confidence intervals when data are generated from Normal(13,400) and Lognormal(2,1) distributions for three sample sizes.

**Fig 2:**
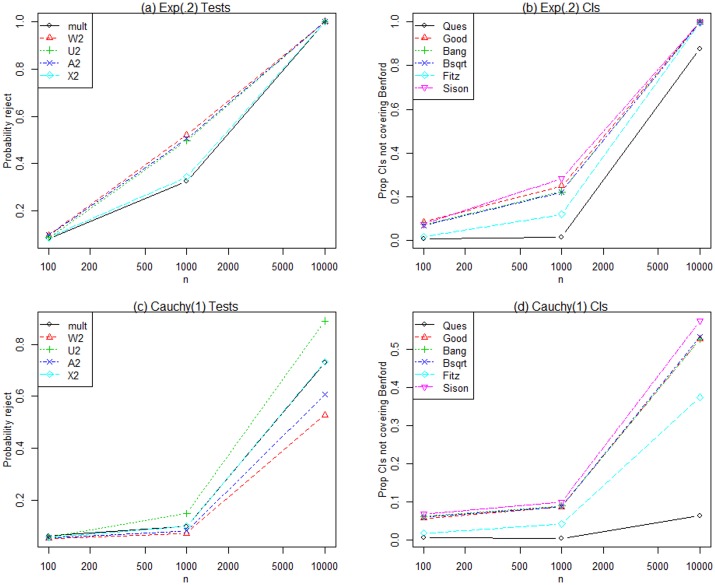
Exponential(.2) and Cauchy(1) results. Simulated power for the tests and simultaneous confidence intervals when data are generated from Exponential(.2) and Cauchy(1) distributions for three sample sizes.

**Fig 3:**
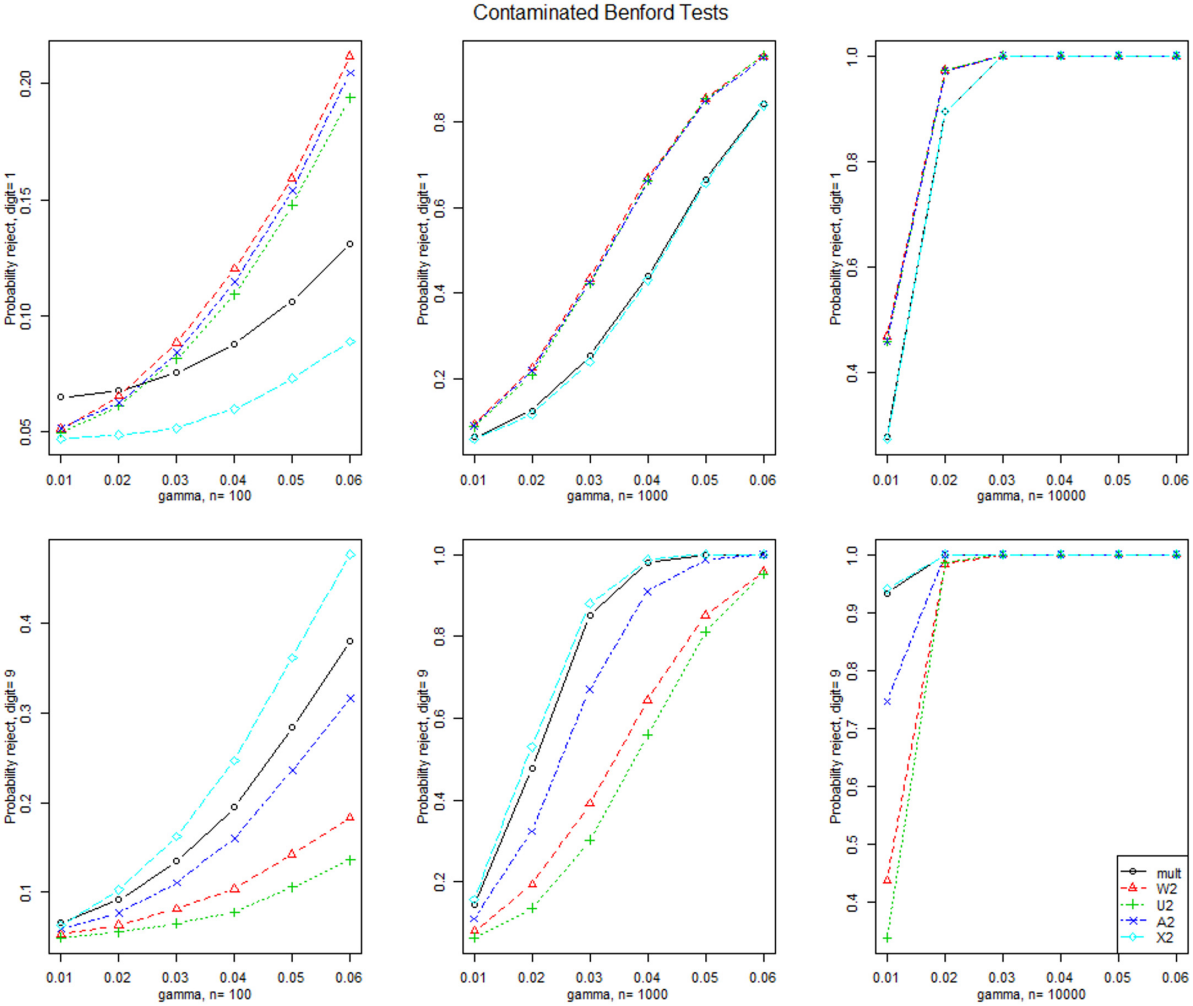
Contaminated Benford distribution test results. Simulated power for the tests when data are generated from the Contaminated Benford distribution where digits 1 and 9 are contaminated by an additive amount *γ* and for three sample sizes.

**Fig 4:**
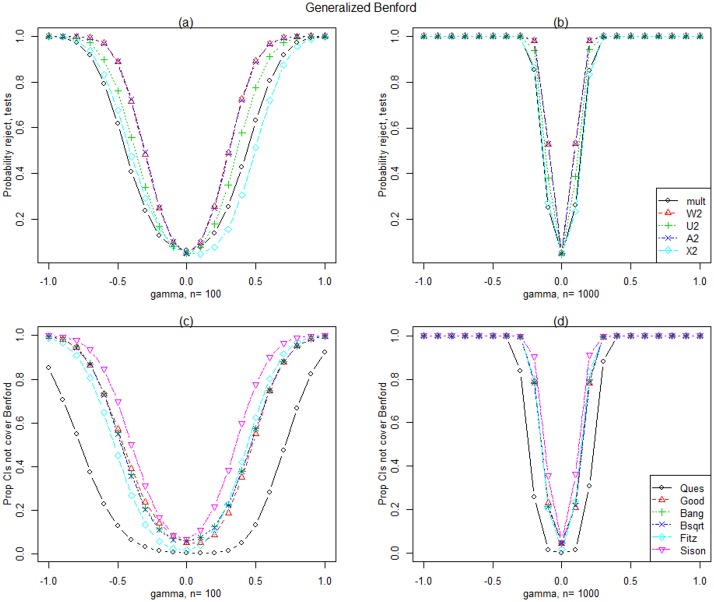
Generalized Benford distribution results. Simulated power for the tests and simultaneous confidence intervals when data is generated from the Generalized Benford distribution with various values of *γ* and for two sample sizes.

**Fig 5:**
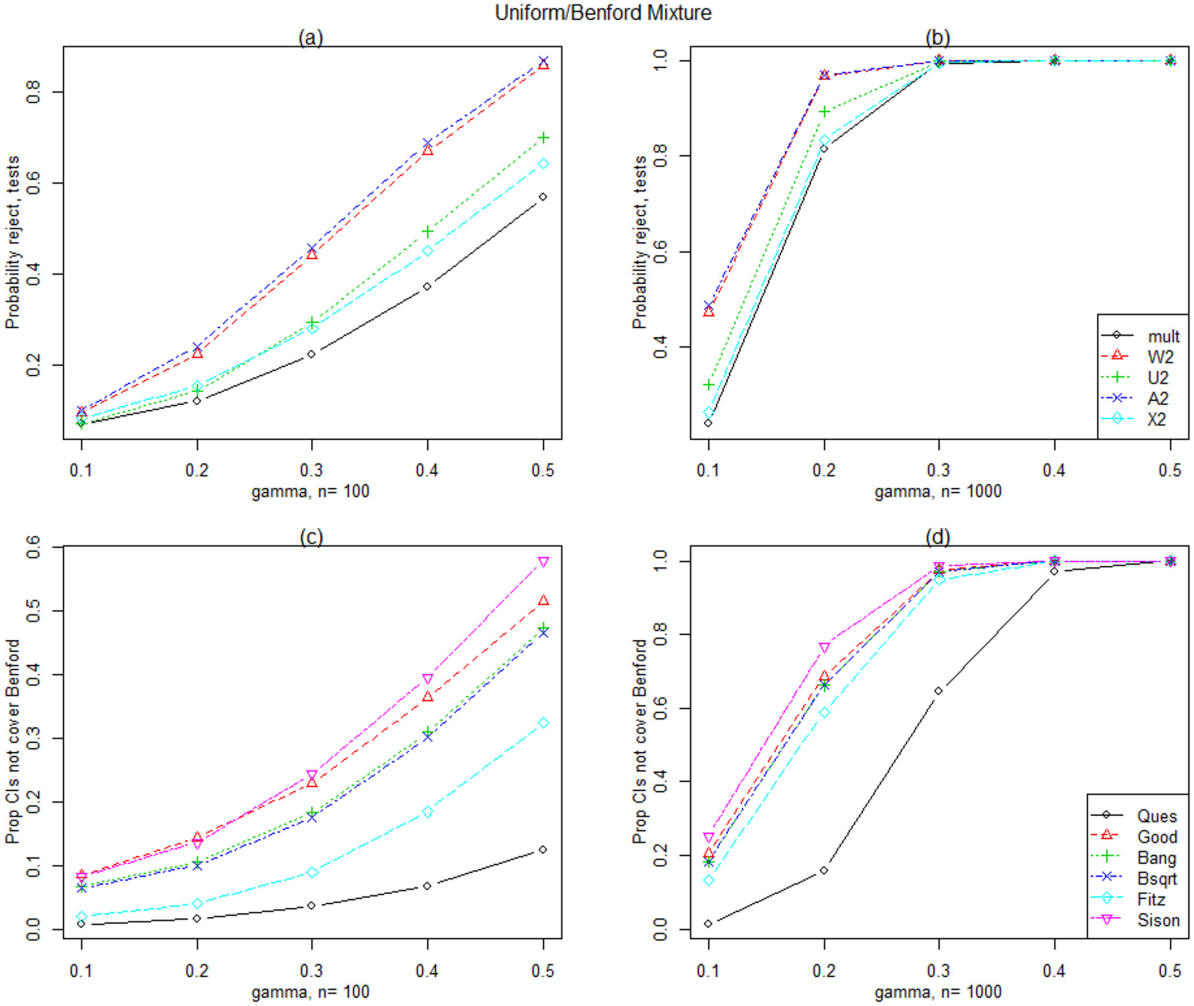
Uniform/Benford mixture distribution results. Simulated power for the tests and simultaneous confidence intervals when data are generated from the Uniform/Benford mixture distribution with various values of *γ* and for two sample sizes.

1. Table 3 shows the proportion of samples of size *n* rejected at the 0.05 level when the generating distribution is Benford. With N = 10,000 replications, the margin of error (2 standard errors) is 0.004, and all test statistics except the LR statistic with *n* = 100 show acceptable size (Type I error rate); that is, the proportions rejected are close to 0.05 when the generating distribution is Benford.
2. We investigated the empirical power, defined as the proportion of *N* = 10,000 samples which reject the null hypothesis of Benford at the 0.05 level, for each of the test statistics and alternative distributions given in Table 2. All test statistics have excellent power for detecting the discrete and continuous uniform alternatives for all *n* and the results are not shown here.
3. Simulated power for the Normal(13,400) is given in Fig 1(a). The results are very similar for Normal(0,1). All statistics have good power for large *n*, and $U_{d}^{2}$ has the largest power for *n* = 100. Fig 1(c) also displays results for the Lognormal(2,1) where none of the statistics have much power for *n* = 100 or even *n* = 1,000, but the CvM statistics, especially $U_{d}^{2}$, have good power to detect Lognormal(2,1) departures from Benford when *n* = 10,000. None of the statistics have power to detect Lognormal(2,9) alternatives to Benford (not shown here) because, as Rodriguez (2004) [10] notes, the first digit distribution of Lognormal(2,9) variates is essentially Benford. Fig 2(a) and 2(c) graphs the simulated power for the Exponential(.2) and Cauchy(1) generating distributions respectively. The CvM and $U_{d}^{2}$ statistics perform better than Pearson’s chi-square and LR statistics for the Exponential(.2) and Cauchy(1) distributions respectively.
4. Fig 3 displays the simulated power for the test statistics when the data is generated from the Contaminated Benford for contamination of the first and ninth digits. The CvM statistics have the greatest power for the first digit contamination and Pearson’s chi-square statistic has the largest power for the ninth digit contamination. Power increases with sample size and all statistics have large power when *n* = 10,000 and the contamination exceeds 0.01.
5. Fig 4(a) and 4(b) display the simulated power for Generalized Benford Eq (4) simulated data for *n* = 100 and 1,000. Note that the Generalized Benford distribution tends to Benford as *γ* tends to 0 and we expect the proportion rejected to be approximately 0.05 when *γ* = 0. $A_{d}^{2}$, $W_{d}^{2}$ and $U_{d}^{2}$ have the largest power, however, for *n* = 10,000, all tests perform very well (results not shown).
6. Results for the Uniform/Benford mixture distributions are given in Fig 5(a) and 5(b) for *n* = 100 and 1,000 since all tests perform well for *n* = 10,000. As the proportion, *γ*, of Uniform in the mixture increases, the power increases for all statistics and as for the Generalized Benford, $A_{d}^{2}$, $W_{d}^{2}$ and $U_{d}^{2}$ have the largest power.

**Table 3:** Simulated size of tests.

Test	*n* = 100	*n* = 1000	*n* = 10,000
LR	0.0614	0.0508	0.0482
$W_{d}^{2}$	0.0497	0.0501	0.0523
$U_{d}^{2}$	0.0483	0.0486	0.0501
$A_{d}^{2}$	0.0487	0.0495	0.0527
Pearson’s *X*^2^	0.0525	0.0504	0.0492

Proportion of N = 10,000 samples rejecting Benford when the true simulated distribution is Benford and *α* = 0.05.

### Results: Simultaneous Confidence Intervals

In this section, we assess the performance of simultaneous confidence intervals for testing comformance with Benford’s Law. We do this by generating *N* = 10,000 samples from the distributions given in Table 2 and observing for each sample whether the nine Benford probabilities all fall within the set of simultaneous intervals computed for that sample.

**Fig 6:**
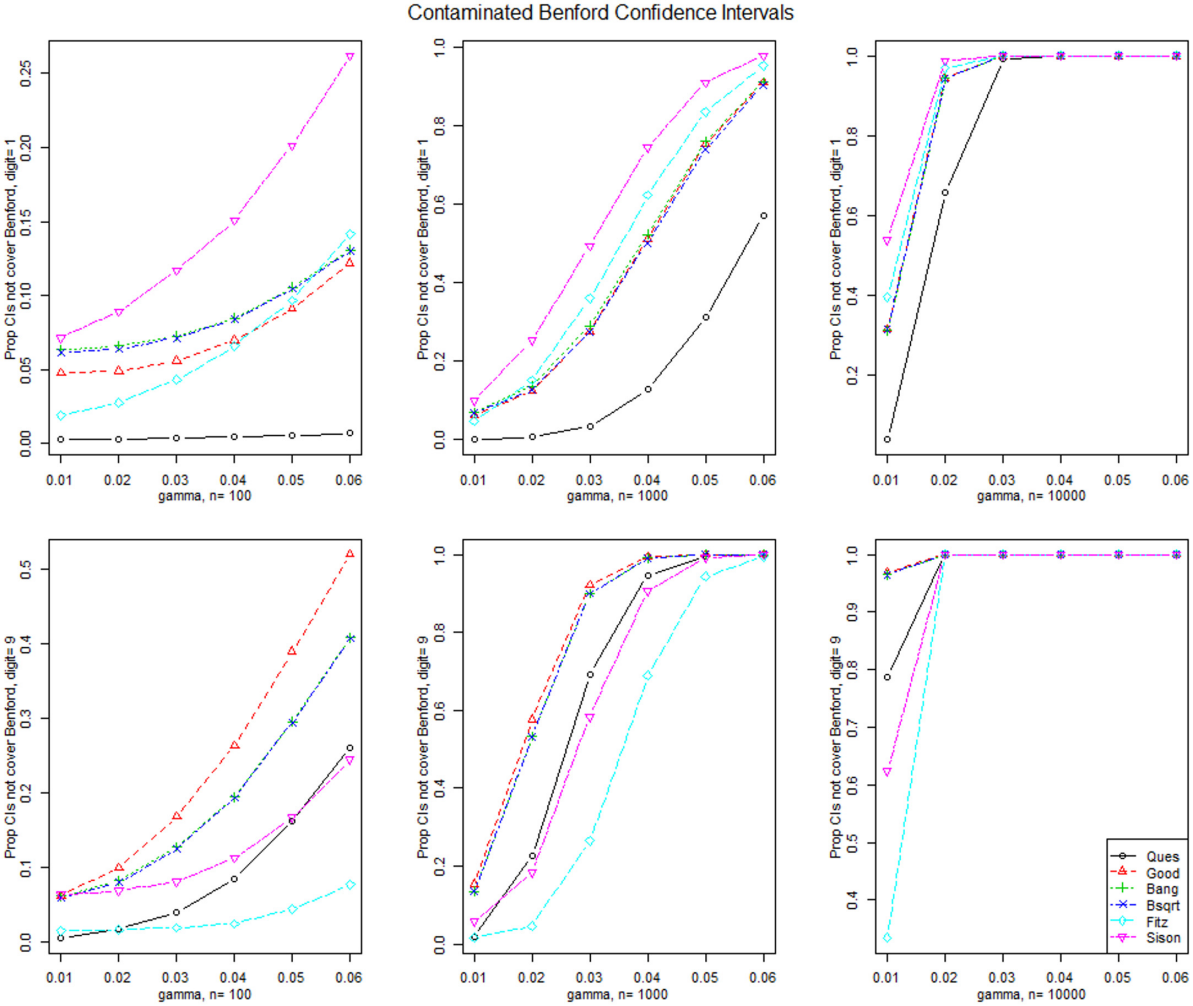
Contaminated Benford distribution CI results. Simulated power for the simultaneous confidence intervals when data are generated from the Contaminated Benford distribution where digits 1 and 9 are contaminated by an additive amount *γ* and for three sample sizes.

1. Table 4 shows the estimated coverage probabilities, that is, the proportions out of the *N* = 10,000 replications such that nominal 95% simultaneous confidence intervals cover the Benford probabilities when the generating distribution is Benford. Note that the approximate margin of error for a coverage probability of 0.95 is 0.004. The Quesenberry intervals are too conservative with coverage proportions much greater than 0.95 under Benford. The Fitz intervals are also quite conservative under Benford and the Sison intervals have a coverage proportion that is marginally too small when *n* = 100. As expected, the Univariate Binomial confidence intervals have very poor (small) coverage proportions under the Benford distribution and we do not consider them in further discussions of power since their size is so far from nominal.
2. To study the power of the simultaneous confidence intervals, we graph the proportion of samples that do NOT simultaneously cover Benford probabilities, or one minus the coverage proportion, since this is analogous to power computed for test statistics. For frequencies generated under the discrete and continuous uniform distributions, all intervals perform well (except Quesenberry) since almost none of the joint sample confidence intervals simultaneously cover the set of Benford probabilities (results not shown here).
3. Results for the Normal(13,400) are shown in Fig 1(b), which are very similar to those for Normal(0,1). All intervals have good power for large *n*, and the Sison intervals have the best power for *n* = 100. Fig 1(d) displays results for the Lognormal(2,1) where none of the intervals have much power for *n* = 100 or even *n* = 1,000, but all but Quesenberry and Fitz have some power to detect Lognormal(2,1) departures from Benford when *n* = 10,000. None of the intervals have power to detect Lognormal(2,9) departures from Benford (not shown here). Fig 2(b) and 2(d) graph the simulated power for the Exponential(.2) and Cauchy(1) generating distributions. The Fitz and Quesenberry intervals do not perform as well as the others for the Exponential(.2) and Cauchy(1) distributions respectively, and the Sison intervals have the greatest power.
4. Fig 6 displays the simulated power for the simultaneous confidence intervals when the data are generated from the Contaminated Benford for contamination of the first and ninth digits. The Sison intervals have the greatest power for the first digit contamination and the Goodman intervals have the largest power for the ninth digit contamination. Power increases with sample size.
5. Fig 4(c) and 4(d) display the simulated power for Generalized Benford Eq (4) generated data for *n* = 100 and 1,000. The Sison intervals have the largest power, however, for *n* = 10,000, all intervals perform very well (results not shown).
6. Results for the Uniform/Benford mixture distributions are given in Fig 5(c) and 5(d) for *n* = 100 and 1,000 since all intervals except Quesenberry perform well for *n* = 10,000. As the proportion, *γ* of Uniform in the mixture increases, the power increases for all intervals, and the Sison and Goodman intervals have the largest power.
7. In comparing the performance of the best simultaneous intervals with the best tests under the alternatives studied, the tests have larger power for detecting departures from Benford than the simultaneous intervals. As expected, both tests and simultaneous confidence intervals have greater power for larger sample sizes and departures from Benford can be detected with large enough samples with the exception of very small contamination. There is not one test statistic that outperforms all others under all of the alternative distributions considered. The CvM statistics generally have the greatest power except for contamination of the larger digits of the Contaminated Benford family. Of the simultaneous confidence intervals, the Goodman and Sison intervals have the largest power in our study.

**Table 4:** Estimated coverage probabilities for Simultaneous Confidence Intervals Law.

Nominal 95% CI	*n* = 100	*n* = 1000	*n* = 10,000
*S*_1_ Ques	0.9967	0.9993	0.9994
*S*_2_ Good	0.9497	0.9538	0.9483
*S*_3_ Bang	0.9399	0.9540	0.9497
*S*_4_ Bsqrt	0.9421	0.9542	0.9487
*S*_5_ Fitz	0.9840	0.9825	0.9812
*S*_6_ Sison	0.9350	0.9495	0.9485
*S*_7_ Univariate Binomial	0.4658	0.6213	0.6404

Proportion of N = 10,000 samples for which the computed 95% simultaneous confidence intervals cover the Benford probabilities when the true simulated distribution is Benford.

## Examples

The following examples demonstrate applications of the tests and simultaneous confidence intervals studied in this paper in assessing conformance of real data to Benford’s Law.

### Genome Sizes

Friar et al. (2012) [25] investigated the distribution of the number of open reading frames (ORFs) for organisms sequenced in the GOLD database (http://www.genomesonline.org/cgi-bin/GOLD/index.cgi) in early 2010. ORFs are subsequences of DNA that are translated into proteins. The authors provided biological arguments as to why they felt the number of ORFs in an organism should be distributed according to Benford’s Law and indeed they found confirmation that the data for the 106 Eukaryotes sequenced in the database conformed to Benford’s Law.

**Fig 7:**
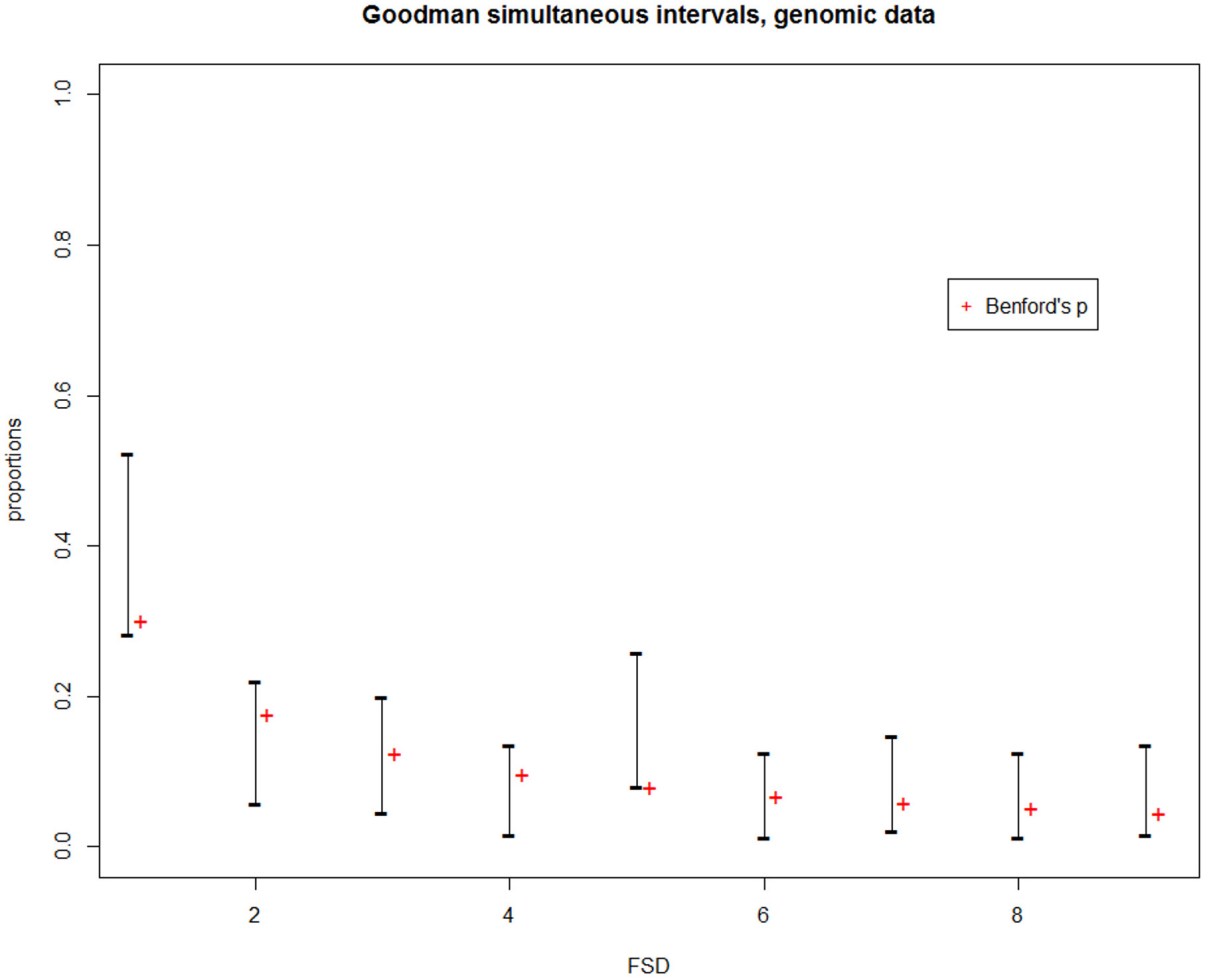
Goodman simultaneous confidence intervals for the genomic data. Vertical line segments denote the Goodman simultaneous confidence intervals computed from the Genomic data in Table 5. The red crosses are positioned at the Benford probabilities.

We have attempted to replicate Friar’s findings using the 2013 GOLD database. In the summer of 2013, the GOLD database held completed sequences for 121 Eukaryotes with their corresponding number of ORFs and total genome sizes. Table 5 displays the first digit observed, relative frequencies and Goodman simultaneous confidence interval values. Table 6 lists p-values for the tests studied in this paper and Fig 7 displays the Goodman simultaneous confidence intervals. $U_{d}^{2}$ is consistent with Pearson’s chi-square and the LR test, all rejecting the hypothesis of Benford at the *α* = 0.05 level. From Fig 7 and Table 5, we note that the frequency of the first digit 5 is larger than expected under Benford, however examination of Fig 7 indicates that it is quite close to it and the difference can be deemed practically insignificant.

**Table 5:** Observed digit frequencies and Goodman simultaneous confidence intervals for genomic data.

Digit	Frequency/Proportion	95% CI lower	95% CI upper	Benford *p*	Cover Benford
1	48/0.397	0.2831	0.5226	0.3010	yes
2	14/0.116	0.0572	0.2202	0.1761	yes
3	12/0.099	0.0462	0.2000	0.1249	yes
4	6/0.050	0.0170	0.1360	0.0969	yes
5	18/0.149	0.0803	0.2592	0.0792	no
6	5/0.041	0.0129	0.1246	0.0669	yes
7	7/0.058	0.0214	0.1472	0.0580	yes
8	8/0.041	0.0129	0.1246	0.0512	yes
9	9/0.050	0.0170	0.1360	0.0458	yes

**Table 6:** P-values for tests of the null hypothesis of Benford’s Law for genomic data.

Test	*n* = 121
LR	0.023
$W_{d}^{2}$	0.126
$U_{d}^{2}$	0.039
$A_{d}^{2}$	0.140
Pearson’s *X*^2^	0.018

### Rodriguez Data

Rodriguez (2004) [10] analyzes 10 financial datasets which we re-analyze using the tests and simultaneous confidence intervals proposed. The series are: net income (NI) and betas (Betas) from the Disclosure Global Researcher SEC database; the annual market rates of return (Mkt Return) from Ibbotson Associates’ Stocks, Bonds, Bills, and Inflation yearbooks; the gross national product (GNP) from the 1998 World Bank Atlas; the group of initial public offering (IPO) data, initial price (IPO Price), number of shares (IPO Shares), and total dollar value (IPO Value) by a group of firms; daily Dow Jones Industrial Average (DJ) index values from America Online’s internet portal and their rates of return (deltaDJ/DJ) and the daily changes of the index (deltaDJ). [Note that the values for Pearson’s chi-square statistics for the IPO Shares and Values in Table 3 of [10] are incorrect and should be 49.6 and 20.6 respectively.]

**Fig 8:**
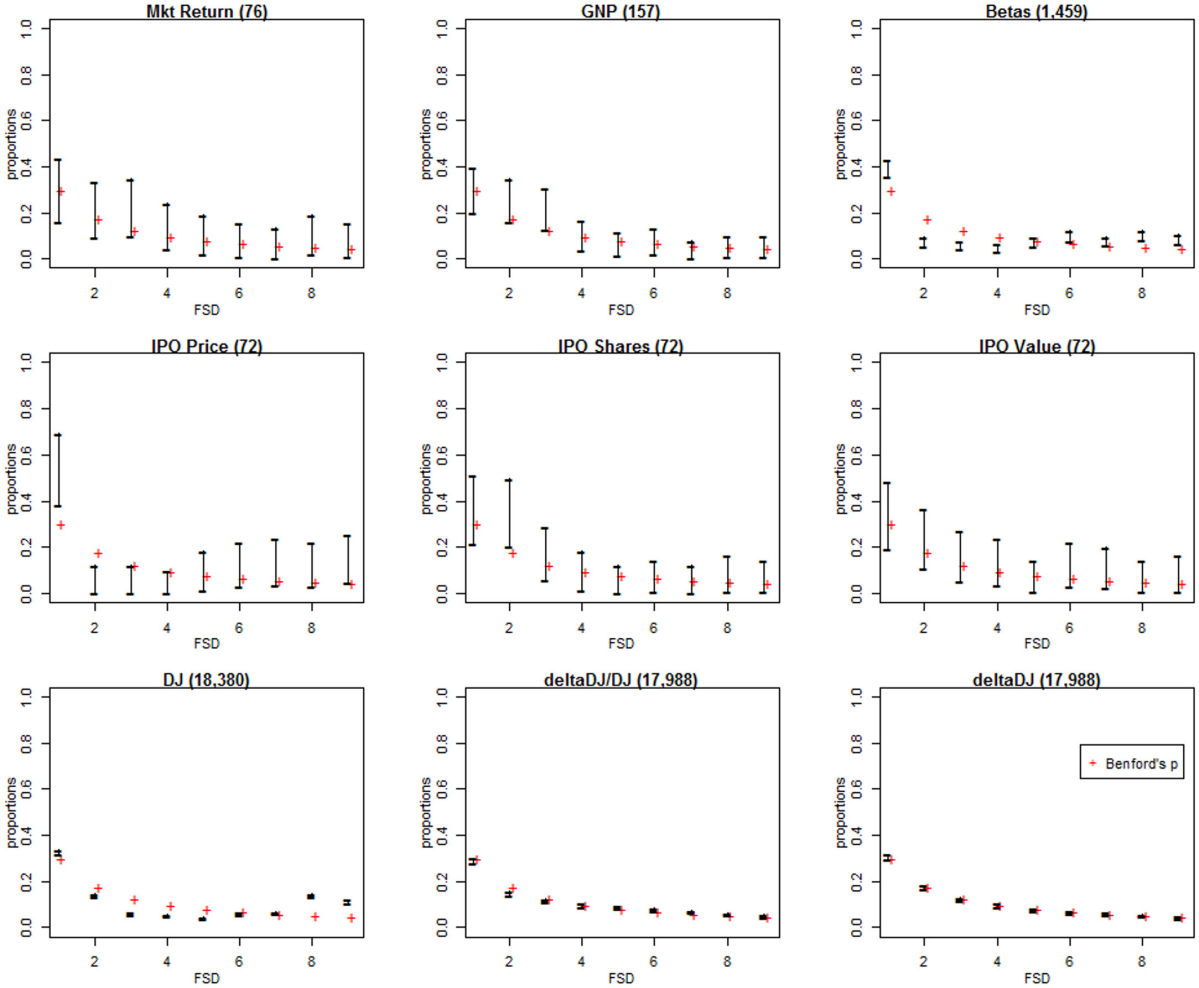
Goodman simultaneous confidence intervals for the Rodriguez data. Vertical line segments denote the Goodman simultaneous confidence intervals computed from the Rodriguez data. The red crosses are positioned at the Benford probabilities. The sample size for each data set is given in brackets in the heading.

Table 7 lists p-values for the CvM and Pearson’s chi-square tests of the hypothesis of Benford as well as indicators of simultaneous coverage of the Benford probabilities by the simultaneous confidence intervals presented in this paper. The test results for the CvM statistics are qualitatively similar to those of Pearson’s chi-square, although $U_{d}^{2}$ is more sensitive, yielding smaller p-values than the Pearson’s chi-square test. For the simultaneous confidence intervals, only the Goodman and Sison simultaneous intervals yield results that are consistent with the test statistics for all datasets. Fig 8 displays the Goodman intervals for nine of the datasets. The intervals are drawn as vertical lines and the red crosses are the Benford probabilities. The widths of the interval estimates clearly display the precision in the confidence interval estimates which is a function of the sample size. The graphs provide clear indications of which digits in the data sets are not consistent with Benford, wherever the crosses do not intersect the vertical lines. We note that GNP and deltaDJ/DJ are not statistically consistent with Benford, however, from the graph, they appear to be practically consistent with Benford since the Benford probabilities are very close to the intervals.

**Table 7:** Tests and simultaneous intervals results for the Rodriguez data.

	P-values				Simultaneous CI coverage of Benford, 1 = yes					
Source (number)	$W_{d}^{2}$	$U_{d}^{2}$	$A_{d}^{2}$	*X*^2^	Ques	Good	Bang	Bsqrt	Fitz	Sison
NI (6,364)	0.334	0.091	0.327	0.293	1	1	1	1	1	1
Mkt Return (76)	0.607	0.384	0.662	0.630	1	1	1	1	1	1
GNP (157)	0.015	0.001	0.014	0.008	1	0	1	1	1	0
Betas (1,459)	0.000	0.000	0.000	0.000	0	0	0	0	0	0
IPO Price (72)	0.000	0.000	0.000	0.000	0	0	0	0	0	0
IPO Shares (72)	0.001	0.000	0.002	0.008	1	0	0	0	0	0
IPO Value (72)	0.660	0.828	0.734	0.843	1	1	1	1	1	1
DJ (18,380)	0.025	0.000	0.004	0.000	0	0	0	0	0	0
deltaDJ/DJ (17,988)	0.000	0.000	0.000	0.000	0	0	0	0	0	0
deltaDJ (17,988)	0.188	0.180	0.217	0.547	1	1	1	1	1	1

P-values for tests of the null hypothesis of Benford’s Law and 95% simultaneous confidence interval coverage for Rodriguez data. A 1 = yes for the simultaneous confidence intervals coverage indicates that all 9 digit intervals cover Benford’s probabilities.

## Discussion

In this paper we proposed and evaluated methods of testing conformance with Benford’s Law. From the simulation study, we observed that Pearson’s chi-square test does not have the greatest power under all alternatives and that the discrete CvM statistics often perform very well. The simulation study also confirmed that separate 100(1 − *α*)% binomial confidence intervals reject the hypothesis of Benford too often for truly Benford data, and they should not be used for this problem. The analyses of the genomic and financial data led to findings that were consistent with those of the simulation study.

As a result of our study, we make the following recommendations:

1. To assess conformance with Benford’s Law, investigators should perform statistical tests; the CvM statistic $U_{d}^{2}$ is recommended and if contamination is expected in the larger values of the first significant digit, Pearson’s chi-square statistic.
2. Visual inspection of data is crucial for any dataset and we recommend that simultaneous confidence intervals are useful for understanding the nature of departures from Benford’s Law. They are also a useful tool for understanding the precision inherent in the data. The Goodman and Sison simultaneous intervals perform best in our study; if computational resources are an issue, then we recommend that the Goodman simultaneous intervals be computed and plotted.

The work presented here applies to the first significant digit. It is extended to the first *m* > 1 digits in Wong (2010) [26]. Asymptotic power approximations are provided in Lesperance (2015) [27] which an investigator can use to perform sample size calculations to ensure that a study is adequately powered. R code for both is available.
